# Does egg donation for mitochondrial replacement techniques generate parental responsibilities?

**DOI:** 10.1136/medethics-2017-104400

**Published:** 2017-10-25

**Authors:** César Palacios-González

**Keywords:** gene therapy/transfer, reproductive medicine

## Abstract

Children created through mitochondrial replacement techniques (MRTs) are commonly presented as possessing 50% of their mother’s nuclear DNA, 50% of their father’s nuclear DNA and the mitochondrial DNA of an egg donor. This lab-engineered genetic composition has prompted two questions: Do children who are the product of an MRT procedure have three *genetic* parents? And, do MRT egg donors have parental responsibilities for the children created? In this paper, I address the second question and in doing so I also address the first one. First, I present a brief account of mitochondrial diseases and MRTs. Second, I examine how MRTs affect the numerical identity of eggs and zygotes. Third, I investigate two genetic accounts of parenthood and MRT egg donation. Fourth, I explore three causal accounts of parenthood and MRT egg donation. My conclusion is that, under the appropriate circumstances, MRT egg donors are parentally responsible for the children created under genetic accounts of parenthood and under causal accounts of parenthood.

## Introduction

Children created through mitochondrial replacement techniques (MRTs) are commonly presented as possessing 50% of their mother’s nuclear DNA (nDNA), 50% of their father’s nDNA and the mitochondrial DNA (mtDNA) of an egg donor.^i^ This lab-engineered genetic composition prompts two questions, among others: Do children who are the product of an MRT procedure have three genetic parents? And, do MRT egg donors have parental responsibilities for the children created?

The academic discussion that is most closely related to the above questions is based on whether MRT egg donation should be anonymous or not.^1–6^ This discussion was primarily prompted because the Nuffield Council on Bioethics (NCoB) report on MRTs,^7^ and the UK’s Human Fertilisation & Embryology Authority 2015 document *Mitochondria replacement consultation: advice to Government*^8^ described how some respondents argued that given that mitochondria are not responsible for conferring personal characteristics, beyond health, then the egg donor should be anonymous. In the end, the UK’s regulation on MRTs followed the Department of Health Response Document’s^9^ recommendations, which held a line similar to that of the previously mentioned respondents, and made MRT egg donation anonymous.^10^

Here, I distance myself from the anonymity debate and instead investigate if an MRT egg donor is parentally responsible for the created child. There are multiple ways in which I could explore this question, and exploring whether MRT egg donors are parentally responsible on each account of parental responsibilities would require much more space that is available here. Instead, I explore MRT egg donation according to two of the most prominent accounts of parental responsibilities: genetic accounts of parenthood (henceforth genetic accounts) and causal accounts of parenthood (henceforth causal accounts). Even if we maintain that monistic accounts of parenthood are bound to fail, such as Bayne and Kolers do, exploring these particular parenthood accounts is useful given that any pluralistic account ‘ought to be broad enough to grant parenthood to [causal], genetic, gestational, custodial and intentional parents’.^11^

This paper adds to the current ethical debate on MRTs in several ways. First, it further critically examines the mainstream position on the relation between mitochondria and parenthood. Second, it tackles in a systematic way the philosophical question if MRT egg donors are parentally responsible for the created children. Third, it opens the door to carry on future research on MRTs and lesbian motherhood. Finally, by showing that under certain circumstances MRT egg donors are parentally responsible for the children created we are in a better position to formulate adequate policies regarding the egg-donation process for MRTs.^ii^

### Mitochondrial diseases and MRTsiii

mtDNA diseases occur when problems within the genes of the mitochondria prevent them from producing the levels of energy that cells need to work properly. They are a group of neuromuscular diseases that can have mild to devastating effects. They cause, for example, heart and major organ failure, dementia, stroke, blindness, deafness, infant encephalopathy and premature death.^12^ Mitochondria are inherited via the maternal line.^iv^ Pathological mutations in the mtDNA can be present either in all mitochondria (homoplasmy), or only in some mitochondria (heteroplasmy).

Recently, two MRTs^v^ have been developed in order to help women with mtDNA diseases to have genetically related children absent such conditions: maternal spindle transfer (MST) and pronuclear transfer (PNT). In MST, assisted reproductive techniques are used to obtain eggs from the intending mother and a healthy donor. The chromosomes from the donor’s oocyte and the intending mother’s oocyte are then extracted. While the donor’s chromosomes and the intending mother’s enucleated oocyte are discarded, the intending mother’s chromosomes are transferred to the now enucleated donor’s oocyte.^vi^ Afterwards, the reconstructed oocyte is fertilised in vitro and then transferred to the intending mother or a surrogate.^7 13^

In PNT two zygotes are created in vitro. One of them is created with the intending parents’ sperm and oocyte (or a sperm from a donor), and the other one with a donated oocyte and the father’s (or donor’s) sperm. After fertilisation, and during the first 24 hours, the maternal and paternal pronuclei are removed from both zygotes. The enucleated cell that was produced with the intending mother’s oocyte and the pronuclei that were contained in the cell produced with the donor’s oocyte are discarded. Subsequently, the intending parents’ (or donor’s and intending mother’s) pronuclei are transferred to the enucleated cell produced with the donor’s oocyte. The reconstructed zygote is then transferred to the intending mother or a surrogate.^7 14^ In both techniques, the donor’s healthy mitochondria will be passed down via the maternal line to subsequent generations, if everything goes as expected.

### Numerical identity and MRTs

In order to discuss causal and genetic accounts of parenthood, we need first to understand how MRTs affect the identity of eggs and oocytes. Let us start with the difference between *qualitative* identity and *numerical* identity. Qualitative identity indicates to the fact that two entities share certain properties, things can be qualitatively identical in different degrees. For example, we would say that two phones are qualitatively identical if they were produced by the same company, have the same type of chips, are the same model, etc, but without them being one and the same object. Numerical identity is only held between a thing and itself. For example, Mary Shelly is numerically identical to the woman who wrote Frankenstein, they are one and the same person.

Once we have pointed this out, and before we can ask whether MRTs affect numerical identity or qualitative identity, we need to explicitly state what kind of things eggs and zygotes are. According to Matthew Liao’s account of the Organism View, an oocyte is essentially a cell, and a zygote is essentially an organism.^15–17^

That eggs are essentially cells entails: (1) that they begin to exist when the capacity to regulate and coordinate the various life processes is there; (2) persist as long as there is a continuing ability to regulate and coordinate the various life processes (ie, cellular continuity) and (3) cease to exist when the capacity to regulate and coordinate the various life processes ceases to be.^17^ Zygotes come to be as organisms and cease to exist when they are no longer organisms. A zygote begins to exist when: (1) the capacity to regulate and coordinate the various life processes is there; (2) persists as long as there is ‘organismic continuity’, which is the continuing ability to regulate and coordinate the various life processes and (3) ceases to exist when the capacity to regulate and coordinate the various life processes ceases to be.^17^^-^vii -viii

If we grant the above then we have to accept that both MST and PNT *affect the numerical identity of eggs and zygotes*. They do so because ‘the same coordinating and regulating capacity of the various life processes such as metabolism, growth, differentiation and so on’ does not persist after MST, or PNT, takes place.^17^ It does not do so because mitochondria, and not only nuclear DNA, *are essential* for the coordination and regulation of the different life processes: i) the cytoplasm of egg (or zygote) X, where the mitochondria resides, also contains crucial components for regulating and coordinating the various life processes and ii) there are life processes in the cytoplasm of egg (or zygote) X that the nucleus of egg (or zygote) X does not (at least have full) control.^17^

This means that when we enucleate egg (or zygote) X we disrupt its cellular (or organismic) continuity, and thus when we transfer the nuclear material to the enucleated egg (or zygote) Y *a new coordinating and regulating capacity comes to b*e and thus we create a numerically distinct egg (or zygote) Z. What is relevant for our discussion is that this particular MST egg (or PNT zygote) would not have existed but for MST and PNT taking place.^xi^ Now, someone could argue that under the Organism View an MRT results in a *qualitative change* rather than a numerical one. According to the objector, the numerical identity of the egg (or zygote) does not change because it is not necessary that the ‘same’ regulating capacity persists, but rather that ‘a’ regulating capacity does. The problem with this objection is that, in Eric Olson’s words, an organism ‘persists just in case its capacity to direct those vital functions that keep it biologically alive *is not disrupted* (emphasis added)’.^18^ When such regulating capacity is destroyed (during the MRT process), the organism, *qua* organism, is destroyed, and thus a new numerically distinct organism comes into being after the MRT process. The only way in which *the same* numerical egg (or zygote) could persist is if the enucleation process was promptly reversed.

### Genetic accounts of parental responsibilities

The news cycle around the birth of the first baby product of MST was full of headlines that included phrases like: ‘three parent baby’ or ‘three person baby’.^19–21^ The underlying assumption is that children who are the product of MRTs do have *three genetics parents*, but do they?

According to John Harris *they do not*: ‘Although children might be confused if they are told that they have three genetic parents, only a very confused person would think—let alone say—any such thing’.^4^ The NCoB has also stated that ‘it is the view of the Working Group that mitochondrial donation does not indicate, either biologically or legally, any notion of the child having either a "third parent", or "second mother"’.^22^ Now, Harris argues his view on two grounds: mitochondria make up <1% of the total DNA, and they do not confer personal features (save for the susceptibility to avoid disease and suffering). Thus, for him, in order to be a genetic parent one needs to provide more genetic material than the total percentage of mtDNA one, and the genetic material that one provides must confer personal features (eg, skin colour). John Appleby has named these two claims about genetic contribution: the quantity claim and the quality claim.^1^ Neither Appleby nor Harris have investigated how these claims interact with each other. For example, is one a genetic parent if one only provides 2% of the total nuclear genetic material, but such material is *solely* responsible for the child’s facial features (supposing there are such ‘face’ genes)?

On the other side of the debate, we find those who assert that children who are the product of an MRT procedure *do have* three genetic parents. For example, Rebecca Dimond has claimed that ‘focusing on biology alone suggests that all babies born through these techniques would be triparental’.^23^ According to her, this is the case because there is transmission of DNA, in this case mtDNA. Others who have endorsed this view are Jacques Cohen and Mina Alikani,^24^ and Françoise Baylis.^25^ Now, in order to explore this issue in more depth let us turn to what does it mean to be a genetic parent, by investigating genetic accounts.

According to genetic accounts, the genetic link that exists between a parent and her child grounds parental responsibilities. Parental responsibilities, in their more basic sense, should be understood as the nurturing, caring and raising responsibilities that a competent adult has towards an infant. The ‘parental genetic link’ can be understood in at least two different ways. First, A possesses a parental genetic link to B if half of A’s genetic material is present in B. Let us call this the ‘fractional account’. Second, C possesses a genetic link to D if C’s genetic material was derived from D’s genetic material. Let us call this the ‘derivation account’.

The main problem with the fractional account is that half of A’s genetic material is also present in B’s identical twin E, and thus according to it both B and E are equally parentally responsible for A. Given the absurdity of the former, we can confidently conclude that the fractional account is false and that *merely* sharing half of one’s genetic material with someone *is not a sufficient condition* for establishing parental responsibilities.^x^ It is also the case that sharing half of one’s genetic material *is not a necessary condition* for establishing parental responsibilities, as evidenced by adoption cases.

The derivation account maintains that one is parentally responsible for a child if the child’s genetic makeup was *materially derived* from one’s genetic makeup.^xi^ This account, as Austin identifies, can solve the identical twin problem because it ‘contains the concept of causation’.^26^ E is not parentally responsible for A since A was (partially) derived from B’s genetic material. Even though the former seems promising, we must specify that *mere* genetic derivation *is not a sufficient condition* for establishing parental responsibilities, because if it were the case then the following absurd scenario, let us call if ‘theft’, would be true: F has some eggs stored in an in vitro fertilisation (IVF) clinic. G breaks into the clinic and steals F’s eggs; G fertilises them with his sperm and then enters into a surrogacy agreement after which baby H is born. F is parentally responsible for H because H was genetically derived from F. Given this problem, we can modify the genetic/derivation account so that it can adequately deal with ‘theft’. Genetic/derivation account*: the parental genetic-link that exists between A and B grounds parental responsibilities if the act that gave rise to the genetic derivation was one that B consented to in an informed way.

At this point, it might be tempting to think that according to the genetic/derivation account*, MRT egg donation does not establish parental responsibilities. It could be tempting to think so given that in ‘standard’ genetic derivation cases (ie, human sexual reproduction), one member of the couple provides 50% of the nuclear material and the other one the other 50% and all the mtDNA. Reaching this conclusion would be a mistake because the concept of genetic derivation does not necessarily entail that the derivation must happen as it ‘naturally’ occurs in humans. Consider the following hypothetical case: a scientist genetically modifies four zygotes so when they grow into adults their gametes will only contain 25% of the normal human nuclear material. At some point during their adulthood, these four people decide to have a child; they call the same scientist and she helps them create an embryo. Three of them provide 75% of the nuclear material and the other one provides 25% of the nuclear material and all the mtDNA. In this scenario, the four people would be parentally responsible for the child, since the child *was derived* from genetic material that they consented to provide. The former conclusion would stand even if we modify the above case so that two people provided 40% of the nuclear material each and the other two provided 10% each plus the mtDNA. And it would even stand, according to the derivation account, if 100 people donated 1% of the nuclear material and another one all the mtDNA.

If what I have said above is correct then according to the genetic/derivation account*, an MRT egg donor would be parentally responsible for the created child, given that the child is genetically derived from her, in that she provided a percentage of the total genetic material. At this point, I must emphasise that the percentage of derived genetic material is inconsequential; what is important is the genetic derivation which will be a cause of the child’s existence. Someone could object to my conclusion that it merely relies on the quantitative aspect of DNA, and that what matters in genetic derivation is the *qualitative aspect* of the derived genes.

According to this alternative interpretation of the genetic/derivation account*, what grounds parentally responsibilities is the genetic derivation that confers personal characteristics (eg, appearance and psychological traits) to the created child. As previously said, the mainstream position is that mitochondria do not confer personal characteristics and therefore MRT egg donors are not parentally responsible for the created child. There are two problems with this objection when applied to MRTs. First, it seems probable that mitochondrial function determines certain personal characteristics, as Reuven Brandt asserts: ‘There is a growing body of research associating specific mtDNA variants with particular phenotypic traits including personality, psychological disorder and propensity for developing degenerative neurological diseases’.^2^ Now, even if it turned out that there is no relation between mtDNA variants and *non-pathological personality* traits it must be obvious that health, including bodily health, is a personal characteristic, as Annelien Bredenoord *et al* assert when counterfactually comparing someone with, and without, a mtDNA disease: ‘a person without a mtDNA disease will have a different life experience, a different biography and perhaps also a different character’.^27^ Advocates of the ‘personal characteristics’ position need to explain why health is not a personal characteristic. Second, and most important, in standard human reproductive scenarios we would maintain that both progenitors are parental responsible for a created child even if we discovered that only one of them provided the genes that determined personal characteristics. For example, if it were the case that the sperm provided only non-personal characteristics.

Even though I have concluded that under the genetic/derivation account* an MRT egg donor is parentally responsible for the created child, this account, as a unified account of parental responsibilities, is flawed given that it is unable to assign parental responsibility in certain cases, for example, in the case of ‘artificial gametes’. A researcher has been able to create human eggs and sperm from scratch, in the sense that she created them by using organic matter that did not originate in human organisms or cells. Let us specify that the genetic material of each gamete is a completely novel combination. Now imagine that she produces an embryo with these gametes and transfers it to herself, and that later a baby is born. Confronted with this case we have two options: a) we accept that no one is parentally responsible for such a child because the gametes were not derived from a human, which is absurd or b) we accept that the scientist has parental responsibilities towards the child. If we accept the latter, then it becomes even more clear that *causation* plays a large role in determining parental responsibilities. Thus, let us turn to causal accounts of parenthood.

### Causal accounts of parental responsibilities

In the most general sense, a causal account holds that X is parentally responsible for Y if X caused Y to exist. The origin of this responsibility is grounded on the fact that we are *morally responsible* for what we cause, and in reproductive scenarios we cause the existence of a vulnerable needy being that requires sustained care and nurturing. Failure to provide, or see that others provide, such care would cause pain and harm for which we would be morally responsible. As Henry Sidgwick states, ‘For the parent, being the cause of the child’s existing in a helpless condition, would be indirectly the cause of the suffering and death that would result to it if neglected’.^28^ Given the breadth of causal accounts in the next subsections I will only focus on three of them and examine whether MRT egg donors are parentally responsible for the created children.

### Mere causal account of parenthood

A mere causal account maintains, as previously said, that X is parentally responsible for Y if X caused Y to exist. Causation here should be understood counterfactually: if A had not happened, B would not have happened. In order to illustrate this account let us imagine the following scenario, that we shall call ‘sex’: imagine a couple who freely decides to have sex and that as a result of this begets a child. According to the mere causal account, both members of the couple are parentally responsible for the child, in that if they had not had sex when they had it then the child would not have existed.

Let us apply this account to an MRT egg donation case. Suppose that couple A relies on PNT for having a child who is not afflicted by a mtDNA disease, let us call this child B. In this scenario, the egg that was used to produce the zygote with healthy mitochondria was donated by woman C. Now, if woman C had not donated this particular egg then child B would not have existed. This is so because the numerical identity of B *depends* on the transfer of the intending parents’ pronuclei into the enucleated zygote produced with C’s egg, as previously explained. If, alternatively, we had used D’s egg then a *numerically distinct* child, child E, would have been created. We can conclude that the MRT egg donor, under a mere causal account, has parental responsibilities towards B, in that B would not have existed if C had not donated that specific egg.

The mere causal account has been criticised because it is overly reaching, in that it assigns parental responsibilities to too many agents. For example, imagine that the couple in ‘sex’ decides to beget in the ‘Lovely Hotel’. Unfortunately for them, the only way to get to it is by bus and there is only one bus service every day to the hotel and back. Suppose that the couple rides the bus, gets to the hotel and begets child F. In this scenario, the bus driver would also be parentally responsible for F. She would also be so because F would not have existed but for her driving the couple to the Lovely Hotel.

The mere causal account has also been criticised because it provides the wrong results in cases of *proximal causation*. For example, imagine a scenario that we will call ‘lab confusion’: a man goes to his doctor because he thinks he has a low sperm count. The doctor asks for a semen sample and sends it to a lab. In the lab, a technician accidentally uses this sample in an IVF procedure and an embryo is produced. The embryo is transferred to a woman and child G is born. According to the mere causal account, the man who thinks he has a low sperm count is parentally responsible for G. This is because G would not have existed but for him providing a sperm sample to his doctor. Given these two salient problems with the mere causal account, it has been deemed as an inadequate account of parenthood, prompting some to propose revised versions of it.

### Candidate parenthood

Giuliana Fuscaldo has proposed an amended causal account of parenthood that she calls ‘candidate parenthood’. According to her, ‘an account of how moral responsibility for children is generated should be consistent with at least the standard views about causation, consequences and moral responsibility’.^29^ Such an account seems reasonable as it is true that *we are not morally responsible* for everything we are causally connected to.

There are two commonly accepted conditions for establishing moral responsibility: foreseeability and freedom. Freedom: ‘we are not responsible for actions that are unavoidable, or in situations where we are not free to do otherwise’.^29^ Foreseeability: we can be held accountable for the consequences of our actions ‘if a reasonable person would have reason to expect that they might occur’.^29^ But, what does ‘free’ mean and who is a ‘reasonable person’? Fuscaldo asserts that for an action to be free either an agent could have done otherwise or, if no alternative was possible, she reflectively sanctioned or appropriated her actions. Defining who is a reasonable person, on the other hand, is more complicated and would divert us from the present discussion. Nonetheless, we can confidently assert that all reasonable people understand a) that the creation of a child might be a consequence of us providing gametes for a reproductive endeavour and that b) a child might be created by an heterosexual couple having unprotected vaginal sex. At this point, we can assert that: ‘*we are morally accountable for the intended and unintended reasonably foreseeable consequences of our free actions*’.^29^ When we apply these two conditions of moral responsibility to reproductive scenarios, we have what Fuscaldo calls candidate parenthood: ‘any (free) action that reasonably foreseeably results in the birth of a child generates responsibilities for that child’.^29^ On this account, ‘standard’ gamete donation, where there is informed consent, generates parental responsibilities. It does so in that the creation of a child is a reasonably foreseeable consequence of the donation of sperm or eggs.

Let us now examine an MRT egg donation in relation to candidate parenthood. According to candidate parenthood, the woman with whose healthy egg an MRT procedure is carried out can either be parentally responsible for the created child, or not. She *would be* parentally responsible in those cases where it was reasonably foreseeable that the consequence of her free action would be the creation of a child, for example, if she donated her eggs with due informed consent, as would be the standard case in the UK.^10^ On the other hand, she *would not be* parentally responsible in those scenarios where it was not reasonably foreseeable that the consequence of her action would be the creation of a child. Consider the following case: after a cancer diagnosis a woman undergoes the total removal of her reproductive system. Unbeknown to her, some of her eggs are used for an MRT procedure and a child is created. According to candidate parenthood, such a woman *is not* paternally responsible for the created child. This is because it is not a reasonably foreseeable consequence of undergoing this surgery that your eggs will later on be used for such a reproductive endeavour.

Even though Fuscaldo’s candidate parenthood account has many advantages over a mere causal account, in that, for example, it does not assign parental responsibilities to those who acted under duress, it has the downside that it too assigns parental responsibilities to too many actors. For example, imagine a scenario that we shall call ‘A Lovely Ride’, which is a modified version of ‘Lovely Hotel’: in this setup, the couple arrives at the bus station just as the bus is returning from the hotel. The driver tells them that they will have to wait until tomorrow. Suppose that the couple talks the driver into driving them to the hotel. They tell her that they intend to have sex and beget a child that night, and just how much it would mean for them if she could take them. The driver, breaking the rules and in her own free time, freely accepts to give them a ride to the hotel where the couple in fact begets a child. Now, in this scenario the bus driver *is parentally responsible* for the created child; she is so in that the creation of a child is a reasonably foreseeable consequence of her driving the couple to the hotel. This is because the couple explicitly told her what they intended to do and she *freely* took them. That in ‘A Lovely Ride’ the driver is parentally responsible for the child is *highly* counterintuitive.

Fuscaldo replies to the above kind of worry stating that ‘no one is suggesting that IVF scientists or clinicians have duties for all of the children they help to bring about’.^29^ Even if she does not *suggest* this she cannot scape this consequence, since the conditions established by candidate parenthood are attained in such cases. The way out of the conundrum is to argue that parental responsibilities can be transferred or delegated and therefore MRT egg donors, gamete providers, bus drivers and IVF scientists can in fact transfer or delegate theirs.^30^

### A bifurcated causal account of parenthood

Lindsey Porter has proposed another version of a causal account of parenthood, which she calls the ‘bifurcated causal account of parenthood’. According to it, ‘causing a child to come into existence places one in a distinct moral role to which obligation attaches, but does not make one a parent; while occupying the role ‘parent’ also obliges one to one’s child(ren)’.^31^ On this account being a ‘maker’ (ie, the one who causes the child to exist) carries *pro tanto* duties to take on the role of a parent (ie, social/moral parent), but these duties are defeasible.xii Furthermore, being a maker should not be understood *as only limited* to those who provide the gametes with which the embryo is produced, but also comprises other agents who cause the child’s existence, such as, in the case of gamete donation, the intending parents. In order to determine who is a causal agent, Porter assumes John Leslie Mackie’s^32^ conceptualisation of causation, according to which causes are at a minimum INUS conditions: Insufficient but Necessary parts of a condition which is itself Unnecessary but Sufficient.

Now, a maker’s obligation towards a child is ‘roughly, the obligation to make the child’s existence a good one to the extent that one can’.^31^ This entails that makers have an obligation to enter into the role of social/moral parent when those who were supposed to occupy it can no longer do so. Thus, given that gamete donors are ‘makers’, they have morally weighty responsibilities. It must be clear that Porter’s account suffers from the same overinclusiveness problem as Fuscaldo’s one, the only difference being the type of responsibilities that ‘makers’ have.

Let us turn to MRTs. Under the bifurcated causal account of parenthood MRT egg donors are ‘makers’. This is because the egg donation is an insufficient (because we need another egg, or zygote) but necessary (if we change the egg donor a numerically distinct child would be created) part of a condition which is itself unnecessary (the couple could just adopt a healthy child) but sufficient for the child to be created. This being the case, we can confidently assert that, under this account of parenthood, MRT egg donors have defeasible *pro tanto* duties towards the created children when the donation was carried out with informed consent. In other words, an MRT egg donor has a parental moral obligation to ensure the created child is taken care of, and she should act, *ceteris paribus*, as the caring parent if the caring duties are unfulfilled.

## Conclusion

In this paper, I explored whether MRT egg donors are parentally responsible for the children created after an MRT procedure under genetic accounts and causal accounts. Given that this was my only aim, I did not stop to investigate how and under what circumstances should the delegation, or transfer, of parental responsibilities occur. According to the most promising genetic account, the genetic/derivation account*, MRT egg donors are parentally responsible for the created children when the donation occurs with adequate informed consent. Now, two of the three causal accounts of parenthood that I explored yield the result that if adequate informed consent was obtained then MRT egg donors have parental, or maker, obligations towards the created children, and on the third account (the mere causal account) MRT egg donors are parentally responsible even if their eggs were obtained without consent, this fact is further proof that this account is inadequate. Let us finish this paper by stating that those who argue that children created after an MRT do not have three genetic parents, and that MRT egg donors have not parental obligations towards the created children need to revise their positions or come with new arguments for showing this.

## Footnotes

i Three things should be clear. The egg donor provides all the cellular machinery except the nuclear DNA. The total percentage of mtDNA provided by the egg donor can vary depending on the amount of mtDNA carryover that occurs during the MRT.^33 34^ The term ‘egg donor’ here should be understood as referring to people who donate their eggs and people who sell their eggs.

ii In this paper, I am only concerned with the question if MRT egg donors are parentally responsible for the created children. Thus, I will not discuss what are the contents of these parental responsibilities.

iii This section draws from Palacios-González’s work on MRTs.^35 36^

iv Only one case of mitochondrial DNA inheritance via the paternal line has been documented in the academic literature.^37^

v As I have said elsewhere: "Even though the name ‘mitochondrial replacement techniques’ is controversial, I use it because it has secured a place within the academic debate. The name is controversial because the techniques transfer *nuclear DNA* and thus a better term would be ‘nuclear replacement techniques".^36^ Ainsley Newson and Anthony Wrigley have recently proposed and defended the term ‘mitochondrial targeting techniques’.^38^

vi During the chromosome transfer, there can be unintentional carryover of pathological mitochondria. A mtDNA disease could manifest if the pathological mitochondria carryover is substantive.^33 34^

vii Anthony Wrigley *et al* seem to hold that cells and zygotes are essentially their nDNA and therefore that numerical identity follows the nuclear genome.^39^

viii In his work on the Organism View, Liao does not explore on the differences between cells and organisms.

ix An MST egg is the end result of ‘the process of MST’ and a PNT zygote is the end result of ‘the process of PNT’; ‘process’ here entails the enucleation, transfer and reconstitution actions.^36^

x Avery Kolers^40^ and Robert Sparrow^41^ have explored this issue in relation to reproductive cloning.

xi Just as in the fractional account, genetic derivation is not necessary for establishing parental responsibilities.

xii Porter here follows David Archard’s distinction between parental obligations and parental responsibilities. According to Archard, ‘There is an obligation to ensure *that* someone acts as a parent to the child, and there are the responsibilities *of acting as a parent*’.^42^
